# An unusual case report on the possible role of Warfarin in migraine prophylaxis

**DOI:** 10.1186/2193-1801-2-48

**Published:** 2013-02-12

**Authors:** Angelo Russo, Sara Santi, Daniela Gueraldi, Maria De Paola, Fabiana Zani, Luigi Alberto Pini

**Affiliations:** 1 Pediatric Neurology Unit, Modena and Reggio Emilia University, Modena, 41124 Italy; 2 Health psychology, Polyclinic of Modena, Modena, 41124 Italy; 3 Interdepartmental Centre of Headache and Drug abuse, Polyclinic of Modena, Modena, 41124 Italy

**Keywords:** Anticoagulant drugs, Aura, Coagulation, Migraine

## Abstract

**Background:**

Migraine is a complex disease whose physiopathological mechanisms are still not completely revealed.

**Findings:**

We describe an unusual case, not yet described in literature, of a patient who reported migraine remission, but still presented aura attacks, since starting a therapy with Warfarin.

**Conclusions:**

This case report brings out new questions on the role of the coagulation, especially the blood coagulation pathway, in migraine with aura pathogenesis, and on the possibility to use vitamin K synthesis inhibitors, Warfarin or new generation drugs, as possible therapy to use in migraine prophylaxis.

## Introduction

Migraine is a complex disease whose physiopathological mechanisms are still not completely revealed, despite the numerous improvements that have been achieved lately in research (Edvinsson & Uddman 2005). A possible connection between migraine and stroke was described (Weinberger 2007). The risk of developing a stroke is higher in patients suffering from migraine with aura than without aura; a possible explanation for this association could be an hypercoagulable state (Corral et al. 1998; Moschiano et al. 2004). There are few cases reported in literature in which a substantial reduction of migraine attacks is observed during the use of vitamin K antagonists, but in none of these studies a comparison has been done between patients with migraine and migraine with aura episodes (Maggioni et al. 2012). We present an unusual case, not yet described in literature, of a patient who reported migraine remission but still presented aura attacks since starting a therapy with Warfarin. We discuss the possible role of anticoagulant in migraine prophylaxis and the implications of the blood coagulation pathway in migraine pathogenesis.

### Case report

On January 2012, a 31-year-old patient came to the Modena headache outpatients clinic with his mother (54 years old). His mother was diagnosed with migraine with aura, according to criteria of the International Headache Classification (ICH2 2004), complied by the International Headache Society (IHS) and who was not responsive to pharmacological therapy starting from adolescence. His family medical history reported that also his father (60 years old), and his two siblings (his sister, 28 years old, and his brother, 17 years old) were diagnosed with migraine with aura (ICH2 2004), poor responsive to pharmacological therapy. Our patient was the only family member who presented only 4 migraine attacks with visual aura attacks, with the same clinical presentation as his family. These episodes appeared at the age of 16, recurrent monthly and with spontaneous remission. From the interview it emerged that he has been in therapy with Warfarin since he was 17, after a surgical procedure for the substitution of his aortic valve with a mechanical one. The native valve was insufficient because of an untreated rheumatic fever that he had in his childhood. Furthermore, we surprisingly learned that, once he had started Warfarin therapy, approximately once a month he presented visual aura without the following migraine attack. For this reason we decided to re-evaluate the patient after a week. In this occasion we examined all his clinical reports before the cardiac surgery, and we didn’t find any data supporting the hypothesis of a secondary cause for his previous migraine attacks; also, we confirmed that he suffered from migraine with aura, according to ICH2 2004. Also, we didn’t find any abnormality on physical and psychological examination, brain MRI and routine blood tests. Eventually, we examined more carefully the patient regarding his coagulative state. He was evaluated for antithrombin 3, plasminogen, protein C and S, prothrombin time, activated partial thromboplastin time, factor V-Leiden, von Willebrand factor ristocetin cofactor activity, antinuclear antibody, serum lactic and pyruvic acid, lupus anticoagulant, antiphospholipid antibodies, factor II and methylenetetrahydrofolate reductase and homocysteine. We didn’t find anything beyond the normal range, beside his value of INR of 2.64, due to his Warfarin therapy.

## Discussion

Current molecular and functional studies suggest a way to incorporate the different aspects into an integrated hypothesis as neurovascular headaches (Edvinsson & Uddman 2005). There are evidences that a prothrombotic tendency may be involved in migraine pathogenesis, particularly in patients with migraine with aura and patients with migrainous stroke (Weinberger 2007; Corral et al. 1998; Moschiano et al. 2004). In the last 15 years, the possible role of coagulation defects in migraine patients during attacks and in the intercritical phase has partially been investigated, and some alterations have been discovered, such as resistance to activate protein C due to Factor V Leiden mutation, factor II 20210 mutation, factor V 1692 mutation, antithrombin, protein C, and protein S deficiencies, elevated factor VIII levels and homocysteinemia (Maggioni et al. 2012; Hering-Hanit et al. 2001). Furthermore, in literature, many cases are described in which patients reported migraine remission or mayor improvements after starting a Warfarin therapy for a different cause; in these studies it was not evaluated the different patient’s answer in migraine with or without aura (Teber et al. 2011). We present the unusual case, not yet described in literature, of a patient who reported migraine remission but still presented aura attacks with a monthly frequency since starting a therapy with Warfarin after a surgical procedure of aortic valve substitution. The fact that he still presented aura attacks is very important, because coagulation defects are nowadays considered more relevant in the pathogenesis of migraine with aura than without(Edvinsson & Uddman 2005; Weinberger 2007; Corral et al. 1998; Moschiano et al. 2004); moreover, our patient didn’t have any coagulation deficit. This might invite to a careful consideration on the effective role of the blood coagulation pathway on migraine with and without aura pathogenesis. This last hypotesis is reinforced considering the fact that our patient is the only family member who experienced a complete remission of migraine attacks, with only aura episodes. Indeed, the other four components of his family, that in this case we can consider as controls, kept suffering from migraine with aura attacks, and all of them are poor responsive to pharmacological therapy. Given the high ratio between risks and benefits with the dosage that our patient was using to obtain an INR between 2 and 3, several authors suggested to carry on observational studies with Warfarin with dosage that can make patients achieve an INR only slightly beyond 1.2, but in literature the only double-blind-trial with acenocoumarol at low dosage failed to demonstrate the usefulness in migraine prophylaxis (Wammes-van der Heijden et al. 2005). Currently, experiments with new anticoagulant drugs that might both answer on the possible role of blood coagulation pathway on migraine pathogenesis and that might be used as prophylactive therapy are taking place (Ahrens et al. 2010; Mavrakanas & Bounameux 2011). Also changes of global hemodynamic after aortic-valve replacement could be considered on migraine with and without aura outcome. Further studies are needed to assess the exact role of the blood coagulation pathway in the pathogenesis of migraine with and without aura; the role of Warfarin in the improvement of migraine prognosis and the possible different reactions to Warfarin between patients with migraine with and without aura. Also, we can consider the use of other anticoagulant drugs that have an effect on the blood coagulation pathway but with a better pharmacokinetic and pharmacodynamic profile.

### Ethical approval

Consent was obtained from the child’s parents for the preparation and publication of this case report.
